# Supplementary material for: The hypoxia-response pathway modulates RAS/MAPK–mediated cell fate decisions in Caenorhabditis elegans
Source: Life Sci Alliance. 2019 May 24;2(3):e201800255. doi: 10.26508/lsa.201800255 (PMC6536719; doi:10.26508/lsa.201800255)
Supplement: Supplementary file 1 [file LSA-2018-00255_TableS1.pdf]

Supplemental table 2

Sequence Name	Gene Name	Genotype	VI	95% C.I.	P	% Muv	n
K10H10.4	-	*let-60(n1046); egl-9(sa307)*[1]	3.09	3.00 - 3.20	0.005	14	22
		let-60(n1046); hif-1(zh111)[1]	4.28	4.10 - 4.51	0.650	90	44
W07A12.6	*oac-54*	*let-60(n1046); egl-9(sa307)*[1]	3.20	3.10 - 3.35	0.102	30	20
		let-60(n1046); hif-1(zh111)[1]	n.a.	n.a.	n.a.	n.a.	n.a.
Y44A6C.1	-	*let-60(n1046); egl-9(sa307)*[1]	3.18	3.05 - 3.34	0.072	23	22
		let-60(n1046); hif-1(zh111)	n.a.	n.a.	n.a.	n.a.	n.a.
T05B4.2	*nhr-57*	*let-60(n1046); egl-9(sa307)*[3]	3.58	3.38 - 3.77	0.000	69	26
		let-60(n1046); hif-1(zh111)[3]	4.70	4.47 - 4.94	0.901	100	32
T25E12.4	*dkf-2*	*let-60(n1046); egl-9(sa307)*[1]	3.28	3.11 - 3.46	0.199	33	27
		let-60(n1046); hif-1(zh111)	n.a.	n.a.	n.a.	n.a.	n.a.
M01H9.3	-	*let-60(n1046); egl-9(sa307)*[1]	3.27	3.05 - 3.55	0.236	25	20
		let-60(n1046); hif-1(zh111)	n.a.	n.a.	n.a.	n.a.	n.a.
C56E6.2	-	*let-60(n1046); egl-9(sa307)*[1]	3.11	3.00 - 3.24	0.014	13	23
		let-60(n1046); hif-1(zh111)[1]	4.03	3.65 - 4.45	0.302	70	20
F22B5.7	*zyg-9*	*let-60(n1046); egl-9(sa307)*[1]	3.08	3.00 - 3.22	0.006	9	23
		let-60(n1046); hif-1(zh111)	n.a.	n.a.	n.a.	n.a.	n.a.
C01B4.1	*str-257*	*let-60(n1046); egl-9(sa307)*[1]	3.10	3.03 - 3.21	0.008	21	19
		let-60(n1046); hif-1(zh111)	n.a.	n.a.	n.a.	n.a.	n.a.
C29F9.5	-	*let-60(n1046); egl-9(sa307)*[1]	3.04	3.00 - 3.11	0.000	9	23
		let-60(n1046); hif-1(zh111)[1]	4.46	4.07 - 4.86	0.797	86	22
C36A4.9	*acs-19*	*let-60(n1046); egl-9(sa307)*[1]	3.12	3.00 - 3.28	0.034	10	20
		let-60(n1046); hif-1(zh111)[1]	4.10	3.73 - 4.45	0.430	80	20
F45D11.16	-	*let-60(n1046); egl-9(sa307)*[2]	3.17	3.05 - 3.33	0.028	19	21
		let-60(n1046); hif-1(zh111)	n.a.	n.a.	n.a.	n.a.	n.a.
W04E12.4	-	*let-60(n1046); egl-9(sa307)*[2]	3.51	3.33 - 3.71	0.838	55	29
		let-60(n1046); hif-1(zh111)[2]	4.22	3.95 - 4.48	0.007	87	30
F53F4.5a	*fmo-4*	*let-60(n1046); egl-9(sa307)*[2]	3.31	3.14 - 3.52	0.243	38	21
		let-60(n1046); hif-1(zh111)	n.a.	n.a.	n.a.	n.a.	n.a.
K08E3.1	*tyr-2*	*let-60(n1046); egl-9(sa307)*[3]	3.04	3.00 - 3.13	0.281	4	23
		let-60(n1046); hif-1(zh111)[3]	3.96	3.74 - 4.19	0.002	67	55
C01B12.1	*sqt-2*	*let-60(n1046); egl-9(sa307)*[3]	3.20	3.10 - 3.30	0.049	40	20
		let-60(n1046); hif-1(zh111)[3]	4.32	4.08 - 4.55	0.184	95	20
C04F2.1	*srh-79*	*let-60(n1046); egl-9(sa307)*[2]	3.50	3.28 - 3.77	0.756	47	32
		let-60(n1046); hif-1(zh111)[2]	4.61	4.31 - 4.90	0.412	92	26
C13D9.4	*srsx-11*	*let-60(n1046); egl-9(sa307)*[2]	3.45	3.27 - 3.64	0.683	50	28
		let-60(n1046); hif-1(zh111)[2]	4.51	4.09 - 4.89	0.286	86	22
C14E2.5	-	*let-60(n1046); egl-9(sa307)*[2]	3.47	3.18 - 3.80	0.676	36	25
		let-60(n1046); hif-1(zh111)	n.a.	n.a.	n.a.	n.a.	n.a.
C28H8.8	-	*let-60(n1046); egl-9(sa307)*[3]	3.16	3.00 - 3.36	0.784	14	22
		let-60(n1046); hif-1(zh111)[3]	4.35	4.12 - 4.62	0.244	96	26
C50E3.9	-	*let-60(n1046); egl-9(sa307)*[2]	3.48	3.33 - 3.65	0.822	61	33
		let-60(n1046); hif-1(zh111)[2]	4.65	4.31 - 4.98	0.480	90	29
F13B9.6	*cutl-29*	*let-60(n1046); egl-9(sa307)*[2]	3.55	3.33 - 3.79	0.882	57	21
		let-60(n1046); hif-1(zh111)	n.a.	n.a.	n.a.	n.a.	n.a.
F56C3.3	-	*let-60(n1046); egl-9(sa307)*[2]	3.31	3.15 - 3.52	0.262	35	31
		let-60(n1046); hif-1(zh111)[2]	4.35	3.98 - 4.72	0.084	83	23
K02F6.1	-	*let-60(n1046); egl-9(sa307)*[4]	3.03	3.00 - 3.08	0.000	5	20
		let-60(n1046); hif-1(zh111)[4]	4.14	3.91 - 4.34	0.005	86	29
R04D3.1	*cyp-14A4*	*let-60(n1046); egl-9(sa307)*[2]	3.49	3.33 - 3.66	0.794	61	38
		let-60(n1046); hif-1(zh111)[2]	4.98	4.72 - 5.25	0.027	97	30
R52.2	-	*let-60(n1046); egl-9(sa307)*[4]	3.58	3.38 - 3.83	0.050	58	26
		let-60(n1046); hif-1(zh111)[4]	4.93	4.64 - 5.24	0.902	97	29
T05B9.2	-	*let-60(n1046); egl-9(sa307)*[2]	3.07	3.00 - 3.17	0.000	11	27
		let-60(n1046); hif-1(zh111)[2]	4.71	4.40 - 4.98	0.627	97	30
T06G6.2	*sra-24*	*let-60(n1046); egl-9(sa307)*[4]	3.52	3.30 - 3.80	0.870	41	27
		let-60(n1046); hif-1(zh111)[4]	5.15	4.89 - 5.39	0.005	100	23
T07H3.4	*clec-21*	*let-60(n1046); egl-9(sa307)*[2]	3.29	3.11 - 3.52	0.241	28	32
		let-60(n1046); hif-1(zh111)[2]	4.23	3.95 - 4.55	0.008	83	29
Y38C1BA.3	*col-109*	*let-60(n1046); egl-9(sa307)*[2]	3.51	3.31 - 3.73	0.806	46	39
		let-60(n1046); hif-1(zh111)[2]	4.80	4.43 - 5.15	0.757	90	20
Y38H6C.2	*srw-65*	*let-60(n1046); egl-9(sa307)*[2]	3.38	3.22 - 3.58	0.475	44	25
		let-60(n1046); hif-1(zh111)[2]	4.69	4.42 - 4.95	0.576	97	31
CC8.2	-	*let-60(n1046); egl-9(sa307)*	n.a.	n.a.	n.a.	n.a.	n.a.
		let-60(n1046); hif-1(zh111)	n.a.	n.a.	n.a.	n.a.	n.a.
eV	-	*let-60(n1046); egl-9(sa307)*[1]	3.40	3.14 - 3.67	-	38	21
		let-60(n1046); hif-1(zh111)[1]	4.17	3.71 - 4.66	-	68	19
eV	-	*let-60(n1046); egl-9(sa307)*[2]	3.39	3.24 - 3.53	-	41	59
		let-60(n1046); hif-1(zh111)[2]	4.65	4.44 - 4.85	-	98	56
eV	-	*let-60(n1046); egl-9(sa307)*[3]	3.08	3.00 - 3.15	-	15	20
		let-60(n1046); hif-1(zh111)[3]	4.48	4.23 - 4.73	-	95	22
eV	-	*let-60(n1046); egl-9(sa307)*[4]	3.34	3.18 - 3.52	-	43	28
		let-60(n1046); hif-1(zh111)[4]	4.64	4.29 - 4.94	-	100	24
