# Supplementary material for: The hypoxia-response pathway modulates RAS/MAPK–mediated cell fate decisions in Caenorhabditis elegans
Source: Life Sci Alliance. 2019 May 24;2(3):e201800255. doi: 10.26508/lsa.201800255 (PMC6536719; doi:10.26508/lsa.201800255)
Supplement: Supplementary file 2 [file LSA-2018-00255_TableS2.pdf]

Supplemental table 1

Sequence Name	Gene Name
K08C7.5	*fmo-2*
F22B5.4	-
F42A10.4a	*efk-1*
F59B10.4	-
C24B9.9	*dod-3*
F44B9.1a	*dpf-6*
C31G12.2	*clec-245*
Y38E10A.23	-
R10D12.1	-
F56A3.3a	*npp-6*
C34D4.14	*hecd-1*
K04H4.2	-
C04A2.1	*tbc-6*
F02E8.6	*npc-1/ncr-1*
F45D11.1	-
K02E7.6	-
F16G10.10	-
Y48G8AL.6	*smg-2*
C01G6.8a	*cam-1*
F57B9.1	-
F21D12.3	-
T08G3.6	-
Y46G5A.6	-
M05D6.5	-
C34B7.3	*cyp-36A1*
M03A1.1a	*vab-1*
Y55F3BR.4	*lgc-33*
C32D5.12	-
Y19D10A.5	-
C05D12.1	-
C05E4.2	*str-20*
C10G11.3	*srh-51*
C42D4.13	-
C42D4.2	-
C50E10.6	*sre-54*
F20D6.1	-
F25G6.5	*stdh-4*
F57G8.1	*srh-180*
H27D07.6	*srh-87*
R09B5.3	*cnc-2*
R09E10.2	-
R186.6	*mpst-7*
T04A11.10	*sru-17*
T06E4.8	-
T07C5.5	*nhr-26*
T17H7.1	-
W02A2.3	*pqn-74*
ZK938.6	*chil-9*
