# Supplementary material for: The hypoxia-response pathway modulates RAS/MAPK–mediated cell fate decisions in Caenorhabditis elegans
Source: Life Sci Alliance. 2019 May 24;2(3):e201800255. doi: 10.26508/lsa.201800255 (PMC6536719; doi:10.26508/lsa.201800255)
Supplement: Supplementary file 3 [file LSA-2018-00255_TableS3.pdf]

strain number(s)	genotype	reference	remarks
AH13	*let-23(sy1)*	Aroian and Sternberg 1991	outcrossed original strain
AH2927	*rrf-3(pk1426); rde-1(ne219); let-60(n1046); zhEx418[lin-31p::rde-1; myo-2::mCherry]*	Haag et al. 2014	
AH3195, AH3196, AH3198	*let-60(n1046); vhl-1(ok161)*	this study	
AH3201, AH3253, AH3254	*let-60(n1046)*	this study	from cross with *vhl-1(ok161)*
AH3264, AH3265, AH3292	*let-60(n1046); hif-1(ia04)*	this study	
AH3266, AH3267, AH3268	*let-60(n1046)*	this study	from cross with *hif-(ia04)*
AH3270, AH3271	*let-60(n1046)*	this study	from cross with *egl-9(sa307lf)*
AH3340	*let-60(zh122) (CB4856 background)*	this study	
AH3345	*let-60(zh121) (N2 background)*	this study	
AH4425	*rrf-3(pk1426); rde-1(ne219); let-60(n1046); duIs?[elt-2p::rde-1(+); pRF4]*	Schmid et al. 2015	
AH4533	*hif-1(zh111)*	this study	
AH4553, AH4554, AH4565	*let-60(n1046); hif-1(zh111)*	this study	
AH4571-AH4573	*let-60(n1046); egl-9(sa307) hif-1(ia04)*	this study	
AH4576	*unc-119(ed3); let-60(n1046); iaIs38[egl-9p::egl-9::tag, unc-119(+)]*	this study	
AH4577	*let-60(n1046); egl-9(sa307); iaIs38[egl-9p::egl-9::tag, unc-119(+)]*	this study	
AH4588	*lin-45(sy96)*	this study	from cross with *egl-9(sa307lf)*
AH4594	*iaIs38[egl-9p::egl-9::tag, unc-119(+)]*	this study	outcrossed original strain
AH4596	*let-60(n1046); iaIs38[egl-9p::egl-9::tag + unc-119(+)]*	this study	
AH4596	*let-60(n2021); egl-9(sa307); iaIs38[egl-9p::egl-9::tag, unc-119(+)]*	this study	
AH4636	*let-60(n1046); iaEx101[egl-9p::egl-9(H487A)::tag, unc-119(+)]*	this study	
AH4638	*lin-12(n137n720)/hT2[bli-4(e937) let-?(q782) qIs48]*	this study	from cross with *egl-9(sa307lf)*
AH4641	*lin-12(n137)/hT2[bli-4(e937) let-?(q782) qIs48]*	this study	from cross with *egl-9(sa307lf)*
AH4661-AH4663	*let-60(n1046); egl-9(sa307)*	this study	
AH4664-AH4666	*let-60(n1046); sos-1(s1031)*	this study	from cross with *egl-9(sa307lf)*
AH4667-AH4669	*let-60(n1046); egl-9(sa307) sos-1(s1031)*	this study	
AH4671	*lin-45(sy96); egl-9(sa307)*	this study	
AH4698-AH4700	*let-60(n1046); egl-9(sa307); iaEx101[egl-9p::egl-9(H487A)::tag, unc-119(+)]*	this study	
AH4703	*let-60(n2021); egl-9(sa307); iaIs38[egl-9p::egl-9::tag, unc-119(+)]*	this study	
AH4722	*unc-32(e189) lin-12(n137n720)/hT2[bli-4(e937) let-?(q782) qIs48] ; egl-9(sa037)*	this study	
AH4728-AH4730	*dpy-19(e1259) lin-12(n137)/hT2[bli-4(e937) let-?(q782) qIs48] ; egl-9(sa307)*	this study	
AH4763	*dpy-19(e1259) lin-12(n137)/hT2[bli-4(e937) let-?(q782) qIs48] ; iaIs38[egl-9p::egl-9::tag, unc-119(+)]*	this study	
AH4826	*egl-9(sa307); opIs206[hif-1p::hif-1::gfp::hif-1 3'UTR, unc-119(+)]*	this study	
AH4833	*unc-32(e189) lin-12(n137n720)/hT2[bli-4(e937) let-?(q782) qIs48]; iaIs38[egl-9p::egl-9::tag, unc-119(+)]*	this study	
AH4833-AH4835	*hT2[bli-4(e937) let-?(q782) qIs48] / lin-12(n137n720); iaIs38[egl-9p::egl-9::tag, unc-119(+)]*	this study	
AH4886,AH4891	*let-60(n1046)*	this study	from cross with *nhr-57(tm4533)*
AH4890, AH4893,AH4894	*let-60(n1046); nhr-57(tm4533)*	this study	
AH4896-AH4898	*let-60(n1046); egl-9(sa307)*	this study	from cross with *nhr-57(tm4533)*
AH4930-AH4932	*let-60(n1046); nhr-57(tm4533) egl-9(sa307)*	this study	
AH4936-AH4938	*let-60(n1046); hif-1(zh111)*	this study	from cross with *nhr-57(tm4533)*
AH4939-AH4941	*let-60(n1046); nhr-57(tm4533) hif-1(zh111)*	this study	
AH5039	*zhEx605[nhr-57p::nhr-57::nhr-57 3' UTR, unc-119(+)]*	this study	
AH5051, AH5052, AH5053	*egl-9(sa307); zhEx605[nhr-57p::nhr-57::nhr-57 3' UTR, unc-119(+)]*	this study	
AH5200	*arTi85[lin-31p::ERK-KTR-mClover-T2A-mCherry-H2B::unc-54 3'UTR]; nhr-57(tm4533)*	this study	
AH5210	*arTi85[lin-31p::ERK-KTR-mClover-T2A-mCherry-H2B::unc-54 3'UTR]; egl-9(sa307)*	this study	
AH552	*let-60(n1046); saIs14[lin-48p::gfp]*	Berset et al. 2005	
AH5531	*arTi85[lin-31p::ERK-KTR-mClover-T2A-mCherry-H2B::unc-54 3'UTR]; egl-9(sa307) hif-1(ia04)*	this study	
AH5535	*arTi85[lin-31p::ERK-KTR-mClover-T2A-mCherry-H2B::unc-54 3'UTR]; hif-1(ia04)*	this study	
AH5536	*arTi85[lin-31p::ERK-KTR-mClover-T2A-mCherry-H2B::unc-54 3'UTR]; hif-1(zh111)*	this study	
BC1925	*let-60(n1046); sos-1(s1031)*	Chang et al. 2000	
CB1413	*lin-7(e1413)*	Simske et al. 1996	
CB1417	*lin-3(e1417)*	Hwang et al. 2004	
CB1439	*lin-10(e1439)*	Whitfield et al. 1999	
EW15	*bar-1(ga80)*	Eisenmann et al. 1998	
GS8190	*arTi85[lin-31p::ERK-KTR-mClover-T2A-mCherry-H2B::unc-54 3'UTR]*	de la Cova et al. 2017	
MT1216	*egl-9(n586)*	Darby et al. 1999	
MT2124	*let-60(n1046)*	Beitel et al. 1990	
MT397	*lin-2(n397)*	Horvitz and Ferguson 1985	
MT4755	*sem-5(n2019)*	Clark et al. 1992	
MT4866	*let-60(ga89)*	Eisenmann et al. 1997	
MT8022	*lin-1(n304)*	Beitel et al. 1995	
PS427	*lin-45(sy96)*	Han et al. 1993	
SD1059	*gaIs37[lin-31::mpk-1; lin-31::D-mek]*	Lackner and Kim, 1998	
SD573	*gaIs47[lin-31::mpk-1(); lin-31::D-mek()]*	Lackner et al. 1998	
WS4274	*opIs206[hif-1p::hif-1::gfp::hif-1 3'UTR, unc-119(+)]*	Sendoel et al. 2010	
WS4289	*unc-119(ed3); vhl-1(ok161); opIs206[hif-1p::hif-1::gfp::hif-1 3'UTR, unc-119(+)]*	Sendoel et al. 2010	
ZG31	*hif-1(ia04)*	Jiang et al. 2001	
